# Predictive effect of triglyceride-glucose index on clinical events in patients with acute ischemic stroke and type 2 diabetes mellitus

**DOI:** 10.1186/s12933-022-01704-4

**Published:** 2022-12-12

**Authors:** Dong Liu, Kaixuan Yang, Hongqiu Gu, Zixiao Li, Yongjun Wang, Yilong Wang

**Affiliations:** 1 Department of Neurology, Beijing Tiantan Hospital, Capital Medical University, Beijing, 100070 China; 2 China National Clinical Research Center for Neurological Diseases, Beijing, 100070 China; 3 Advanced Innovation Center for Human Brain Protection, Capital Medical University, Beijing, 100070 China; 4 Clinical Center for Precision Medicine in Stroke, Capital Medical University, Beijing, 100070 China

**Keywords:** Ischemic stroke, Type-2 diabetes mellitus, The triglyceride-glucose index, Prognosis

## Abstract

**Background:**

The triglyceride-glucose (TyG) index was significantly related to clinical outcome in patients with cardiovascular disease (CAD) and cerebrovascular disease (CVD). We aim to investigate the association between TyG index and clinical prognosis of acute ischemic stroke (IS) patients with type-2 diabetes mellitus (T2DM).

**Methods:**

Among 19,604 patients with acute IS admitted to the China National Stroke Registry II (CNSRII), 3359 IS patients with T2DM were included in the cross-sectional analysis. The TyG index (calculated by ln [fasting triglycerides (mg/dL) × fasting glucose (mg/dL)/2]) was split into four quartiles. The outcomes included recurrent IS, all-cause death and poor outcome at 1 year were analyzed. The association between the TyG index and adverse cerebrovascular outcomes was assessed by proportional hazards regression analysis.

**Results:**

During 1 year follow-up, recurrent IS, all-cause death and poor outcome occurred in 305 (9.08%), 229 (6.82%) and 443 (47.9%) cases, respectively. Multivariable Cox proportional hazards analyses showed that the risk of incident primary endpoints was associated with a higher TyG quartile. After adjustment for confounding factors, patients with a higher TyG index had an association with IS recurrence (adjusted hazard ratio, 1.41; 95% confidence interval, 0.97–2.03; *P* = 0.048) and all-cause death (adjusted hazard ratio, 1.70; 95% confidence interval, 1.062–2.74; P = 0.028), compared with those in the first quartile at 1 year time follow-up. In addition, there were interactions between TyG index and age (≥ 65), female, hypertensive agents, anticoagulant agents, statins and antidiabetic agents in subgroup analyses, especially patients without taken anticoagulant drugs were significantly related to IS recurrence, all-cause death and poor outcome (P = 0.003, P = 0.006 and P = 0.001, respectively).

**Conclusions:**

TyG index is strongly related to the IS recurrence and all-cause death in acute IS patients with T2DM. This finding indicates that the TyG index might be a potential predictor of clinical outcome for acute IS patients with T2DM.

**Supplementary Information:**

The online version contains supplementary material available at 10.1186/s12933-022-01704-4.

## Introduction

Ischemic stroke (IS) accounted for about 70% of stroke cases is one of the main causes of disability and death disease worldwide [1]. The incidence of IS was 101.3 (91–113.6) per 100,000 population [2, 3]. Many researches have shown that pathological and behavioral conditions are associate with the occurrence of IS. These factors include, but are not limited to, diet, smoking habits, hypertension, hyperlipidemia and hyperglycemia [4]. And, several studies have offered consistent evidence for associations between hyperlipidemia, hyperglycemia and the occurrence of IS [5, 6]. Moreover, considering the damage of hyperglycemia and hyperlipidemia to vascular endothelium, to some extent, IS may be a cerebrovascular complication of type-2 diabetes mellitus (T2DM) and hyperlipidemia. And, previous studies have shown triglyceride (TG) as a main risk factor and diabetes as an independent risk factor of cardiovascular disease (CAD) and cerebrovascular disease (CVD) [7]. The triglyceride-glucose (TyG) index, as a specific index of fasting blood glucose (FBG) and TG, has been proposed as a reliable surrogate marker of cardiovascular risk including systematic arterial stiffness, carotid atherosclerosis, coronary artery calcification, coronary artery stenosis, symptomatic coronary artery disease, hypertension, and so on [8, 9]. Furthermore, growing evidences have indicated that TyG index is related to morbidity and mortality of cerebrovascular disease in the general population and many stroke patient cohorts [10, 11].

Calculating the level of TyG index offered a simple method to analyze the sum of blood glucose and lipid status before vascular atherosclerosis [12]. And, vascular atherosclerotic pathological changes are closely related to cerebrovascular and cardiovascular events [13, 14]. Recently, most studies focus on the level of TyG index in healthy and cardiovascular disease individuals to evaluate the incidence and prognosis of CAD and CVD. Some studies indicated that elevated levels of TyG index were associated with higher risk of coronary atherosclerosis progression and all-cause mortality, which was confirmed by some meta-analysis in coronary heart disease studies [15, 16]. And, TyG index was also associated with an increased risk of stroke recurrence, all-cause mortality, and neurologic worsening in acute IS patients [17, 18]. However, there are few clinical studies on TyG index in T2DM and impaired glucose tolerance especially in T2DM patients with IS. And, about the predictive role of TyG index on clinical events such as IS recurrence, all-cause mortality and poor outcome, in IS patients with T2DM has not been fully studied. Based on the research status, the present study was designed with the aim to estimate the role of TyG index in predicting adverse outcomes in acute IS patients with T2DM in analysis of the China National Stroke Registry II (CNSR II).

## Methods

### Study population

From June 2012 to January 2013, a total of 219 nationwide hospitals were selected in China to provide the study individuals of CNSR II [19]. It was approved by the Ethics Committee at Beijing Tiantan Hospital, Capital Medical University. Patients in this study should meet the following eligibility criteria: (1) aged over 18 years old, under 85 years old; (2) diagnosis of acute IS with a mediation history of T2DM within 7 days; (3) direct hospital admission from emergency departments or physician’s clinics; and (4) sign the informed consent by the patients or their legally authorized representatives. Among 25,018 patients with acute cerebrovascular events enrolled in the CNSR II study, 3359 acute IS patients with T2DM were included in this study, finally.

### Data collections and definitions

Baseline information including demographics, mediation history of taking agents, medical history of IS, myocardial infarction (MI), transient ischemic attack (TIA), hyperlipidemia, hypertension, TG, T2DM, angina, atrial fibrillation (AF), medications during hospitalization and at discharge, and National Institutes of Health Stroke Scale (NIHSS) on admission were collected by trained coordinators via face-to-face interviews at each research center.

Patients' peripheral venous blood samples were collected from the antecubital vein in the first fasting blood samples during stay in the hospital within the first 24 h of admission including TG and FBG. The collection, preservation, and processing of blood samples was in a manner according to the clinical site laboratory’s policies and procedures in each center. Concentrations of TG and FBG on admission were extracted from medical records. T2DM was identified as self-reported diabetes previously diagnosed by a physician, current use of antidiabetic agents or FPG ≥ 7.0 mmol/L or 2-h post-load glucose ≥ 11.1 mmol/L or HbA1c ≥ 6.5% according to the China guideline for T2DM. TyG index was calculated as ln [fasting TG (mg/dl) × FBG (mg/dl)/2], as previously described [15].

### Clinical outcomes and follow-up

Patients were followed up at 1 year after discharge in the study. The detailed procedure of follow-up has been previously published. The outcomes included recurrent IS, all-cause death and poor outcome. Poor functional outcomes were defined as a modified Rankin Scale score of 3–6. Clinical prognosis related to rehospitalization was sourced to the corresponding hospitals for the purpose of ensuring reliable diagnosis during the follow-up. About the suspected recurrent cerebrovascular events without hospitalization, the principal investigator together with the trained research coordinators will make a prognostic adjudication finally.

### Statistical analysis

According to the TyG index, patients were divided into four groups by quartiles. We described continuous variables as medians with quartile ranges and categorical variables as proportions. χ^2^ test for categorical variables and Kruskal–Wallis test for continuous variables for the difference between the 4 groups of TyG index quartiles.

To evaluate the association between the primary outcomes and TyG index quartile, we used Kaplan–Meier survival analysis to evaluate the incidence rate of primary outcome events among groups according to different levels of the TyG index, and discrepancies among groups were evaluated by log-rank test. The relationship between TyG index and recurrent IS, all-cause death, and poor outcome at 1 year after discharge was investigated with Cox proportional hazards models. The first quartile of TyG index was taken as a reference in accordance for the event of recurrent IS, all-cause death and poor outcome. Influence factors associated with outcomes at 1 year after discharge were further analyzed after adjustment for relevant factors. Pearson correlation coefficients were used to evaluate the potential for collinearity between TyG index and covariates of FBG, TC, TG, Low density lipoprotein cholesterol (LDL-C), High density lipoprotein cholesterol (HDL-C), FBG, Glycosylated Hemoglobin, Type A1C (HbA1c) in the baseline. The correlation coefficient > 0.5 was considered as a threshold for collinearity. We also conducted subgroup analyses, including age, sex, and hypertensive agents, anticoagulant agents, statins and analyzed the correlation between the above factors and clinical prognosis. Interactions of TyG index and age (< 65 versus ≥ 65 years), sex (male versus female), and hypertensive agents (no versus yes), anticoagulant agents (no versus yes), statins (no versus yes) at discharge on the clinical prognosis were investigated by using Cox proportional hazards models. The hazard ratio was also adjusted for multiple related confounders in the baseline. A 2-tailed P of < 0.05 was considered statistical significance. All statistical analyses were carried out by SAS software, version 9.3 (SAS Institute, Inc, Cary, NC).

## Results

**Fig. 1:**
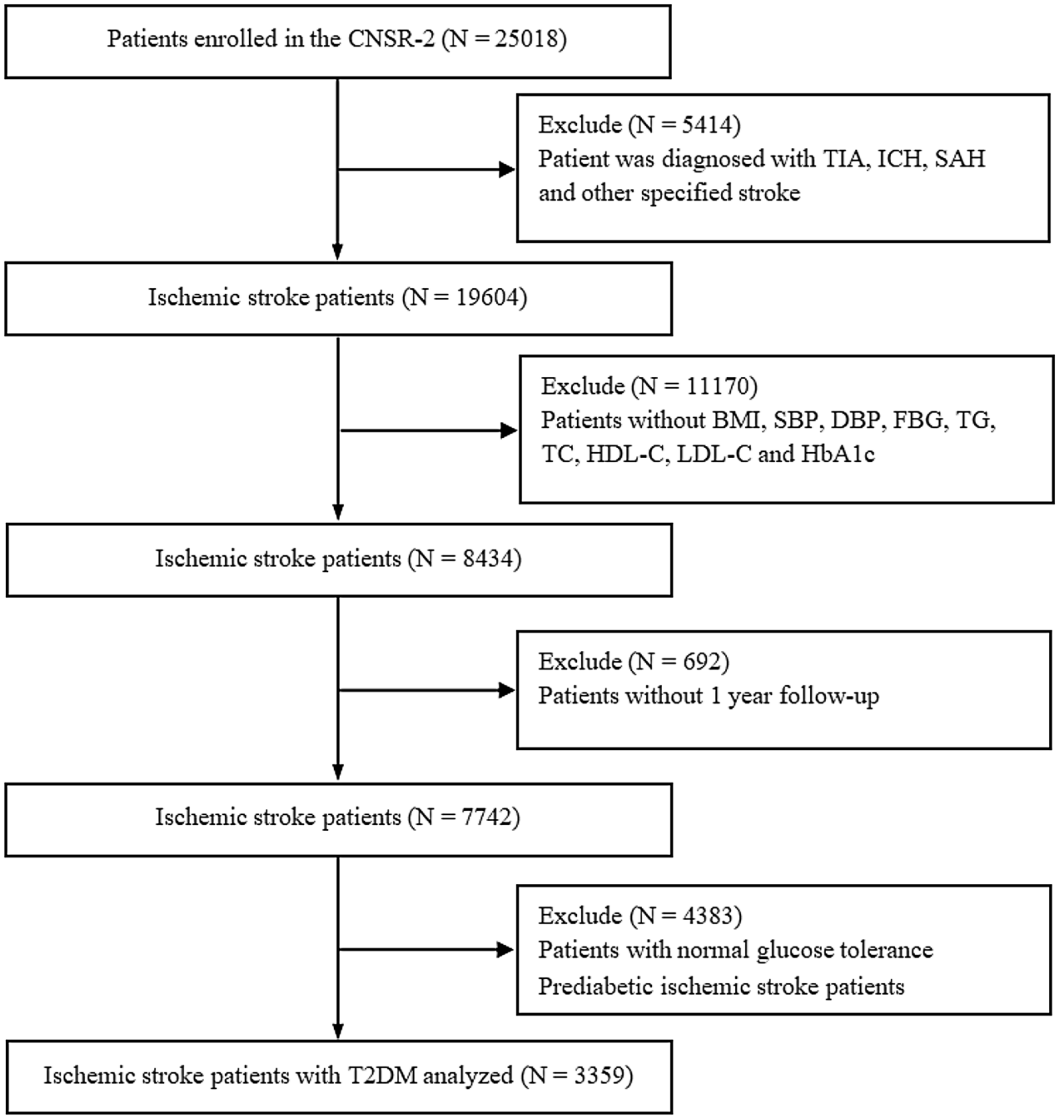
The flowchart of this study. *TIA* transient cerebral ischemic attacks; *ICH* intracranial hemorrhage; *SAH* subarachnoid hemorrhage; *BMI* body mass index; *SBP* systolic blood pressure; *DBP* diastolic blood pressure; *FBG* fasting plasma glucose; *TG* triglyceride; *TC* total cholesterol; *HDL-C* high-density lipoprotein cholesterol; *LDL-C* low-density lipoprotein-C; *HbA1c* Glycosylated Hemoglobin, Type A1C; *T2DM* type-2 diabetes mellitus

In this study, 25,018 stroke patients were enrolled, 5414 of whom diagnosed with TIA, SAH, ICH and stroke other otherwise specified were excluded, 11,170 of whom were excluded without BMI, SBP, DBP, FG, TG, TC, HDL-C, LDL-C and HbA1c. Among the remaining 8434 patients, 692 patients without 1 year follow-up information were also excluded. After further exclusion of 2590 prediabetic IS patients and 1793 patients with normal glucose tolerance, 3359 patients diagnosed with acute IS with a history of T2DM were included in the final analysis (Fig. 1).

### Baseline characteristics

Baseline characteristics of acute IS with T2DM grouped according to quartiles of the TyG index are shown in Table 1. From the result, we can see the mean age of the study subjects was 64.7 ± 11.1 years, and 1990 (59.20%) patients were male. The median TyG index was 1.9 (interquartile range, 1.5–2.4) mmol/L. And, most of patients’ blood glucose and lipids were well controlled. Baseline characteristics of acute IS patients with T2DM stratified by the quartiles of TyG index are shown in Table 2. Patients in the top quartile of TyG index were more likely the male and have slightly higher body mass index (BMI), a lower proportion of high density liptein cholesterol (HDL), a lower proportion of history of taking antihypertensive, statins and anticoagulants before admission, lower proportions of history of IS, TIA, intracranial hemorrhage (ICH), MI, AF and carotid stenosis (CS). Patients in the bottom quartile of TyG were more likely to be older, male, and have slightly lower BMI, lower blood pressure, higher proportions of history of IS, and AF, higher proportions of history of taking antiplatelet, antihypertension, and lipid-lowering agents before admission, and a lower proportion of antihypertension, statins and antidiabetic agents at discharge. Pearson correlation between TyG index and FPG, TC, TG, HbA1c, LDL-C, HDL-C is shown in Additional file 1: Table S1. From the result, we can see TC, TG, LDL, HDL, FBS, HbA1c at discharge were significantly positively correlated linearly with TyG index (*P* < 0.001).

**Table 1:** Characteristics of acute ischemic stroke patients with type-2 diabetes mellitus included according to TyG index quintiles

Characteristics	Overall (N = 3359)	TyG index quartiles				*P* value
		Quarter 1 (N = 839)	Quarter 2 (N = 841)	Quarter 3 (N = 839)	Quarter 4 (N = 840)	
*Patients*						
Age, years	65.0 (57.0–73.0)	68.0 (60.0–76.0)	65.0 (57.0–74.0)	65.0 (57.0–73.0)	61.0 (54.0–69.0)	< 0.001
Male, n (%)	1990 (59.2)	533 (63.5)	511 (60.8)	476 (56.7)	470 (56.0)	0.004
Han, n (%)	3257 (97.0)	815 (97.1)	812 (96.6)	818 (97.5)	812 (96.7)	0.652
BMI, kg/m^2^	24.2 (22.7–26.3)	24.1 (22.2–25.8)	24.4 (22.9–26.3)	24.5 (22.9–26.6)	24.5 (22.9–26.7)	< 0.001
SBP, mm Hg	150.0 (135.0–162.0)	145.0 (131.0–160.0)	150.0 (134.0–160.0)	150.0 (135.0–167.0)	150.0 (135.0–165.5)	0.144
DBP, mm Hg	86.0 (80.0–95.0)	83.0 (80.0–90.0)	85.0 (80.0–94.0)	89.0 (80.0–96.0)	90.0 (80.0–100.0)	< 0.001
NIHSS on admission, median (IQR)	4.0 (2.0–7.0)	4.0 (2.0–6.0)	4.0 (2.0–7.0)	4.0 (2.0–7.0)	4.0 (2.0–6.0)	0.231
Intravenous thrombolysis (r-tPA)	34 (1.0)	15 (1.8)	10 (1.2)	4 (0.5)	5 (0.6)	0.027
*Laboratory examination*						
HbA1c	7.8 ± 2.0	7.0 ± 1.6	7.4 ± 1.6	8.0 ± 2.1	8.8 ± 2.1	< 0.001
TC, mg/dl, mean (SD)	4.8 ± 1.3	4.2 ± 1.1	4.7 ± 1.1	5.0 ± 1.2	5.3 ± 1.4	< 0.001
TG, mg/dl, mean (SD)	2.0 ± 1.5	1.0 ± 0.3	1.4 ± 0.4	2.0 ± 0.6	3.7 ± 2.1	< 0.001
HDL-C, mg/dl, mean (SD)	1.2 ± 0.4	1.2 ± 0.4	1.2 ± 0.3	1.1 ± 0.3	1.1 ± 0.4	< 0.001
LDL-C, mg/dl, mean (SD)	2.9 ± 1.0	2.6 ± 0.9	3.0 ± 0.9	3.1 ± 1.1	3.1 ± 1.0	< 0.001
FBG, mg/dl, mean (SD)	8.6 ± 3.4	6.2 ± 1.8	7.7 ± 2.0	9.0 ± 2.7	11.5 ± 4.0	< 0.001
TyG index, mean (SD)	1.9 ± 0.8	1.0 ± 0.6	1.6 ± 0.1	2.1 ± 0.1	2.9 ± 0.4	< 0.001
*Medication history*						
Antihypertension	1781 (53.0)	446 (53.2)	455 (54.1)	443 (52.8)	437 (52.0)	0.861
Statins	301 (9.0)	101 (12.0)	79 (9.4)	69 (8.2)	52 (6.2)	0.001
Antidiabetics	1657 (49.3)	380 (45.3)	423 (50.3)	418 (49.8)	436 (51.9)	0.044
Anticoagulation	36 (1.1)	13 (1.5)	9 (1.1)	9 (1.1)	5 (0.6)	0.307
*Medical history*						
IS, n (%)	1121 (33.4)	317 (37.8)	299 (35.6)	265 (31.6)	240 (28.6)	0.001
TIA, n (%)	145 (4.3)	38 (4.5)	41 (4.9)	37 (4.4)	29 (3.5)	0.519
ICH, n (%)	77 (2.3)	28 (3.3)	24 (2.9)	15 (1.8)	10 (1.2)	0.013
MI, n (%)	100 (3.0)	31 (3.7)	27 (3.2)	22 (2.6)	20 (2.4)	0.383
AF, n (%)	222 (6.6)	84 (10.0)	58 (6.9)	47 (5.6)	33 (3.9)	< 0.001
CS, n (%)	24 (0.7)	6 (0.7)	8 (1.0)	8 (1.0)	2 (0.2)	0.258
*Medication at discharge*						
Antithrombotics	3005 (89.5)	754 (89.9)	750 (89.2)	749 (89.3)	752 (89.5)	0.968
Antihypertensive agents	1807 (53.8)	417 (49.7)	459 (54.6)	467 (55.7)	464 (55.2)	0.052
Statins	1323 (39.4)	296 (35.3)	344 (40.9)	344 (41.0)	339 (40.4)	0.046
Antidiabetic agents	2019 (60.1)	381 (45.4)	477 (56.7)	540 (64.4)	621 (73.9)	< 0.001
*TOAST subtypes, n (%)*						< 0.001
Cardio embolism	267 (7.9)	104 (12.4)	73 (8.7)	53 (6.3)	37 (4.4)	
Large artery atherosclerosis	136 (4.0)	30 (3.6)	30 (3.6)	43 (5.1)	33 (3.9)	
Small artery occlusion	1662 (49.5)	387 (46.1)	413 (49.1)	403 (48.0)	459 (54.6)	
Other/undetermined	392 (11.7)	106 (12.6)	102 (12.1)	97 (11.6)	87 (10.4)	
Undefined	902 (26.9)	212 (25.3)	223 (26.5)	243 (29.0)	224 (26.7)	

*BMI* body mass index; *SBP* systolic blood pressure; *DBP* diastolic blood pressure; *NIHSS* National Institute of Health stroke scale; *HbA1c* Glycosylated Hemoglobin, Type A1C; *TC* total cholesterol; *TG* triglyceride; *HDL-C* high-density lipoprotein cholesterol; *LDL-C* low-density lipoprotein-C; *FBG* fasting plasma glucose; *IS* ischemic stroke; *TIA* transient cerebral ischemic attacks; *ICH* intracranial hemorrhage; *MI* myocardial infarction; *AF* atrial fibrillation; *TyG index* ln [fasting TG (mg/dl) × FBG (mg/dl)/2]

**Table 2:** HRs (95% CIs) for risk of events in acute ischemic stroke patients with type-2 diabetes mellitus according to TyG index quartiles in this study

	Events, n (%)	Adjusted HR*	*P* value	Adjusted HR†	*P *value	Adjusted HR§	*P *value
1 year follow-up							
*Ischemic stroke recurrence*							
Quintile 1	74 (8.82)	Ref.		Ref.		Ref.	
Quintile 2	69 (8.20)	0.98 (0.70, 1.36)	0.893	0.93 (0.66, 1.32)	0.698	1.01 (0.72, 1.43)	0.949
Quintile 3	95 (11.32)	1.38 (1.02, 1.88)	0.037	1.33 (0.92, 1.91)	0.127	1.41 (0.97, 2.03)	0.048
Quintile 4	67 (7.98)	1.05 (0.75, 1.48)	0.771	0.96 (0.55, 1.67)	0.886	1.06 (0.60, 1.85)	0.843
*All-cause death*							
Quintile 1	63 (7.51)	Ref.		Ref.		Ref	
Quintile 2	54 (6.42)	0.97 (0.67, 1.39)	0.861	1.01 (0.68, 1.51)	0.943	1.14 (0.76, 1.70)	0.531
Quintile 3	69 (8.22)	1.30 (0.92, 1.83)	0.138	1.62 (1.01, 2.61)	0.046	1.70 (1.06, 2.74)	0.028
Quintile 4	43 (5.12)	1.02 (0.69, 1.52)	0.924	1.61 (0.74, 3.52)	0.230	1.67 (0.76, 3.69)	0.201
*Poor outcome*							
Quintile 1	201 (25.57)	Ref.		Ref.		Ref.	
Quintile 2	193 (24.22)	1.05 (0.83, 1.33)	0.701	1.03 (0.78, 1.36)	0.849	1.07 (0.80, 1.43)	0.647
Quintile 3	205 (25.92)	1.16 (0.91, 1.47)	0.230	1.18 (0.85, 1.64)	0.313	1.24 (0.88, 1.75)	0.210
Quintile 4	155 (19.80)	0.99 (0.77, 1.27)	0.916	1.27 (0.77, 2.11)	0.348	1.38 (0.82, 2.33)	0.222

Poor functional outcomes were defined as a modified Rankin Scale score of 3–6

*Adjusted 1, for variables of age and sex

^†^Adjusted 2, adjusted 1 + body mass index (BMI), systolic blood pressure (SBP), diastolic blood pressure (DBP), NIHSS on admission, Glycosylated Hemoglobin, Type A1C (HbA1c), total cholesterol (TC), triglyceride (TG), high-density lipoprotein cholesterol (HDL-C), low-density lipoprotein-C (LDL-C), fasting plasma glucose (FBG)

^§^Adjusted 3, adjusted 2 + intravenous thrombolysis, medical history of ischemic stroke, intracranial hemorrhage (ICH), atrial fibrillation (AF), medication history of Statins, antidiabetics, antihypertension, medication at discharge of antidiabetic agents, statins, antihypertensive agents and TOAST subtypes

### Incidence rate of IS recurrence, all-cause mortality and poor outcome among different groups

During the follow-up period, IS recurrence, all-cause death and poor outcome was occurred in 305 (9.08%), 229 (6.82%) and 754 (22.45%) cases, respectively. Patients who had IS recurrence were 74 (8.82%) in the lowest quartile, 69 (8.20%) in the second quartile, 95 (11.32%) in the third quartile, and 67 (7.98%) in the highest quartile. Furthermore, patients who died included 63 (7.51%) in the lowest quartile, 54 (6.42%) in the second quartile, 69 (8.22%) in the third quartile, and 43 (5.12%) in the highest quartile. The patients who died from IS related events included 13 (3.89%) in the lowest quartile, 12 (2.15%) in the second quartile, 15 (1.57%) in the third quartile, and 26 (1.72%) in the highest quartile. As for poor outcome, 201 (25.57%) in the lowest quartile, 193 (24.22%) in the second quartile, 205 (25.92%) in the third quartile, and 155 (19.80%) in the highest quartile.

**Fig. 2:**
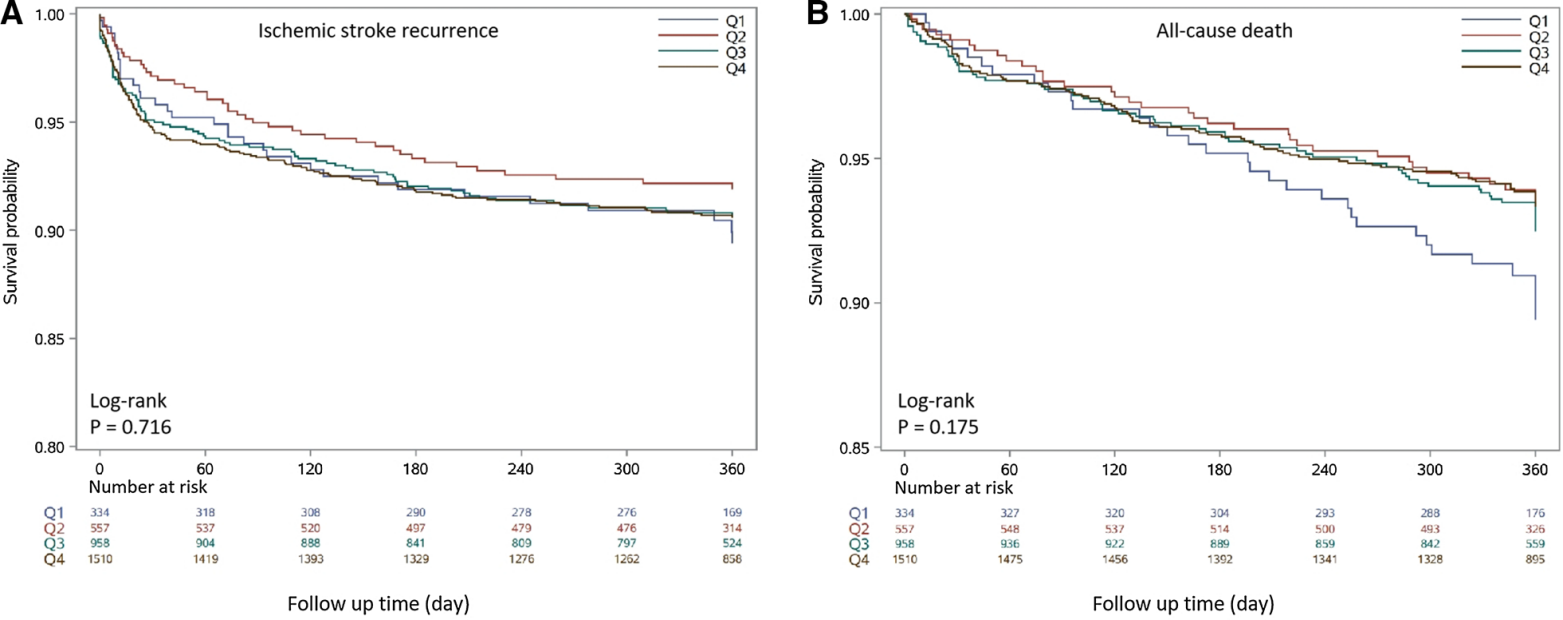
Kaplan–Meier survival analysis curves for ischemic stroke recurrence and all-cause death in acute ischemic stroke patients with type-2 diabetes mellitus according to groups at 1 year follow-up. **A** Landmark analysis for ischemic stroke recurrence. **B** Landmark analysis for all-cause death

The Kaplan–Meier survival analysis curves for assessing the incidence of IS recurrence and all-cause mortality among groups based on the quartile groupings of the TyG index is shown in Fig. 2. Patients with a higher TyG index had a higher risk of recurrent IS. But, there was no statistically significant difference in IS recurrence rate in the four groups (Q1: 8.82% vs. Q2: 8.20% vs. Q3: 11.32% vs. Q4: 7.98%, log-rank *P* = 0.716, Fig. 2A). And, patients with low TyG index had a higher mortality and the reason may be that these patients are older and tend to have other chronic diseases. However, no statistically significant difference with mortality rate in the four groups in one year follow-up (Q1: 25.57% vs. Q2: 24.22% vs. Q3: 25.92% vs. Q4: 19.80%, log-rank *P* = 0.175, Fig. 2B).

### TyG index and clinical outcomes

The prognosis of patients with ischemic stroke at 1 year follow-up in each of the TyG categories was shown in Table 2. Multivariate Cox proportional hazards regression analysis showed that the TyG index, whether considered as a categorical or continuous variable, remained significant in IS recurrence and all-cause death after adjusting for confounders. Three Cox proportional hazards models were established for sensitivity analysis. The higher TyG index was an independent risk predictor of IS recurrence (adjusted hazard ratio, 1.41; 95% confidence interval, 0.97–2.03; *P* = 0.048), compared with those in the first quartile at 1 year follow-up. Furthermore, compared with subjects in the lowest quartile, Patients in the higher quartile of TyG was associated with higher risk of all-cause death within 1 year time follow-up (adjusted hazard ratio, 1.70; 95% confidence interval, 1.062–2.74; *P* = 0.028), compared with those in the first quartile. Furthermore, in order to investigate the prognostic value of tyG index in acute IS patients with abnormal blood glucose, we also further investigated the prognostic value of TyG index in prediabetic patients with acute stroke. About prediabetes patient, Cox proportional risk analysis showed the significant association between TyG index and poor outcome both in adjusted age and sex (HR, 0.72 [95% CI 0.53–0.98] *P* = 0.035) and fully adjusted model (HR, 0.66 [95% CI 0.369–1.11] *P* = 0.016). However, no significant difference was found between TyG index and IS recurrence, all-cause death at 1 year follow-up (all *P* values for interaction > 0.05) (Additional file 2: Table S2).

### Subgroup analysis stratified by TyG index

**Fig. 3:**
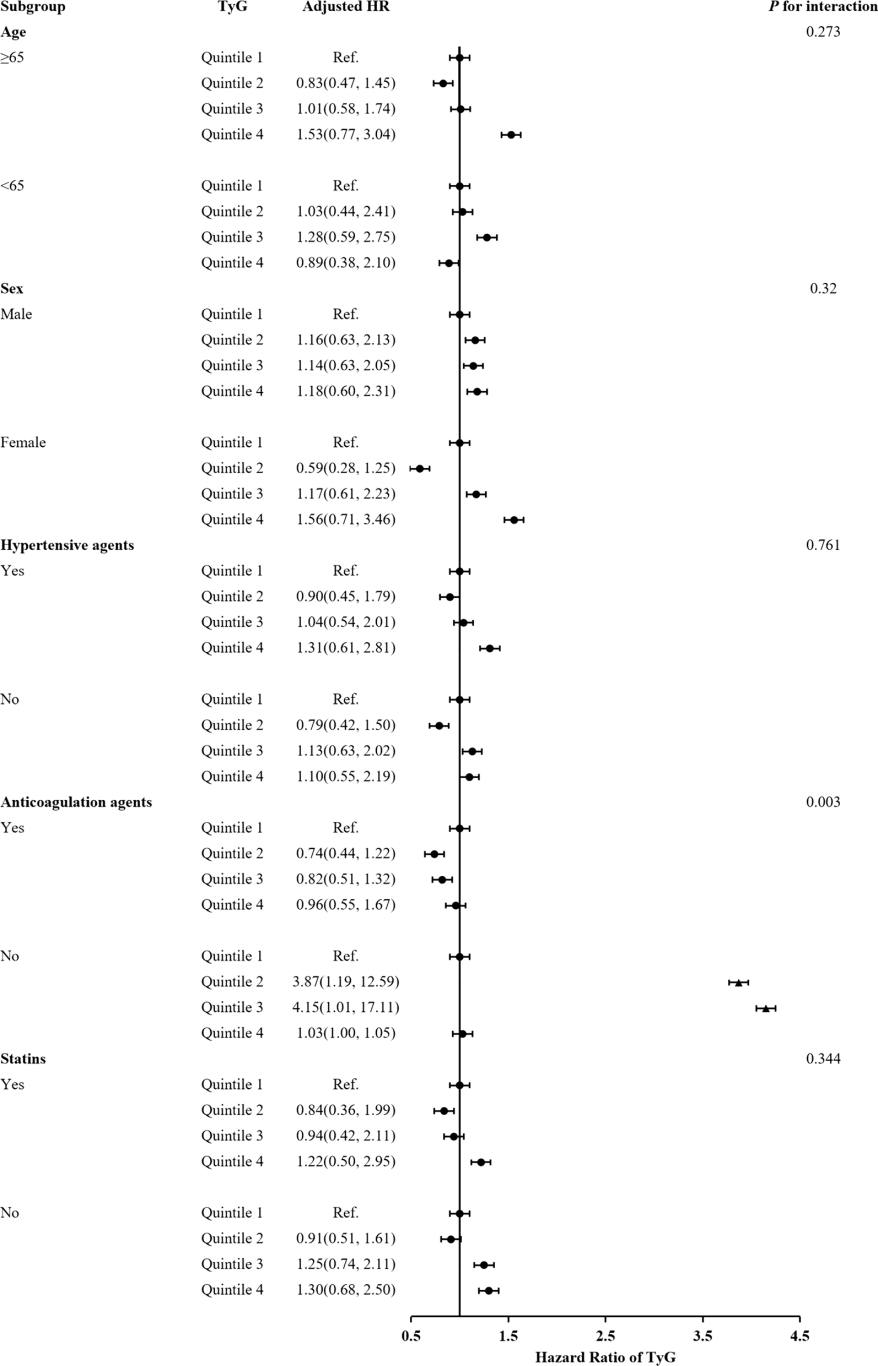
Forest plot of ischemic stroke recurrence in acute ischemic stroke patients with type-2 diabetes mellitus according to different subgroups. Adjusted model included age, sex, body mass index (BMI), systolic blood pressure (SBP), diastolic blood pressure (DBP), NIHSS on admission, Type A1C (HbA1c), total cholesterol (TC), triglyceride (TG), high-density lipoprotein cholesterol (HDL-C), low-density lipoprotein-C (LDL-C), fasting plasma glucose (FBG), intravenous thrombolysis, medical history of ischemic stroke, intracranial hemorrhage (ICH), atrial fibrillation, medication history of Statins, antidiabetics, antihypertension, medication at discharge of antidiabetic agents, statins, antihypertensive agents and TOAST subtypes (▲, P < 0.05)

**Fig. 4:**
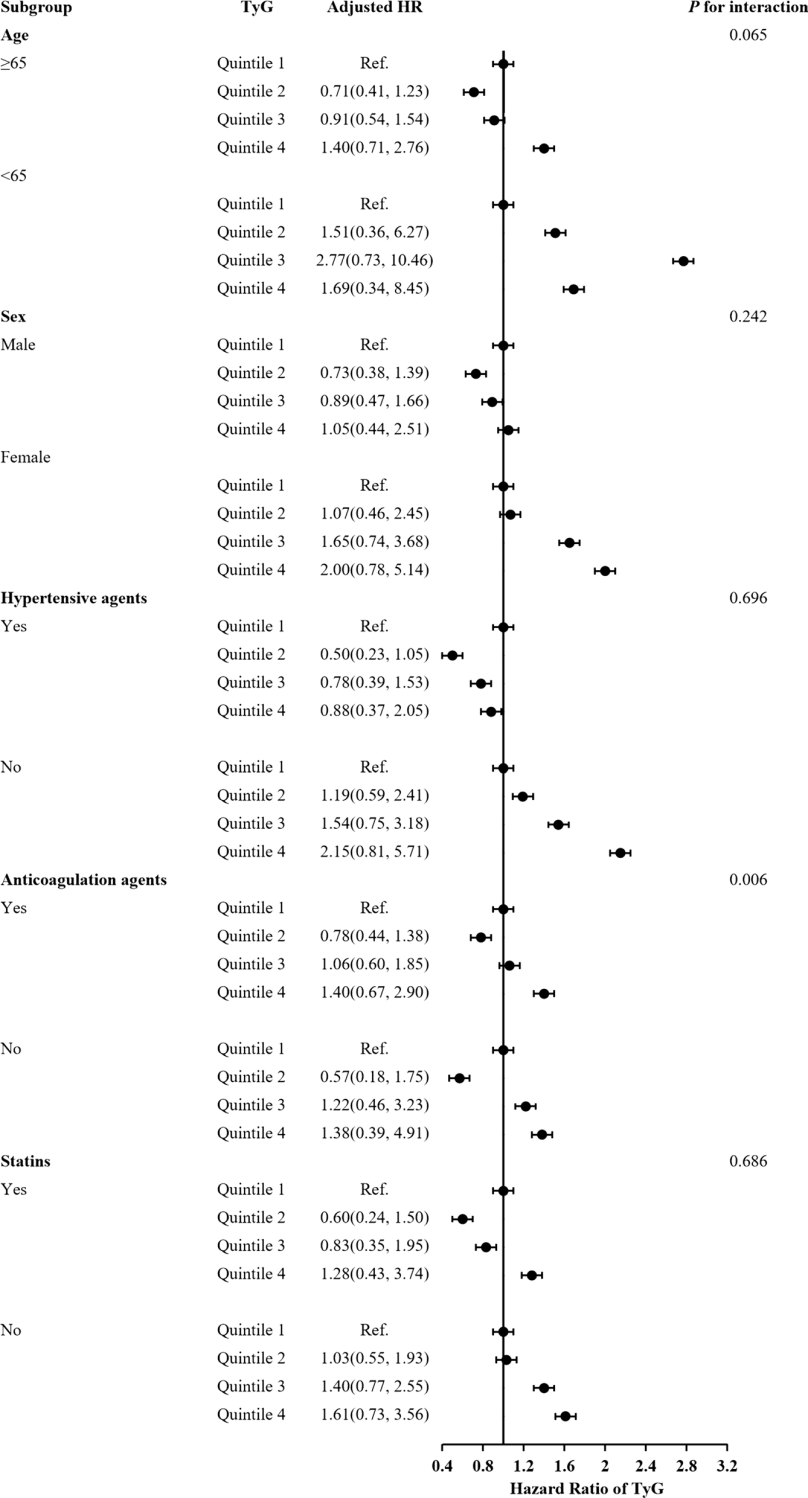
Forest plot of all-cause death in acute ischemic stroke patients with type-2 diabetes mellitus according to different subgroups. Adjusted model included age, sex, body mass index (BMI), systolic blood pressure (SBP), diastolic blood pressure (DBP), NIHSS on admission, Type A1C (HbA1c), total cholesterol (TC), triglyceride (TG), high-density lipoprotein cholesterol (HDL-C), low-density lipoprotein-C (LDL-C), fasting plasma glucose (FBG), intravenous thrombolysis, medical history of ischemic stroke, intracranial hemorrhage (ICH), atrial fibrillation, medication history of Statins, antidiabetics, antihypertension, medication at discharge of antidiabetic agents, statins, antihypertensive agents and TOAST subtypes

**Fig. 5:**
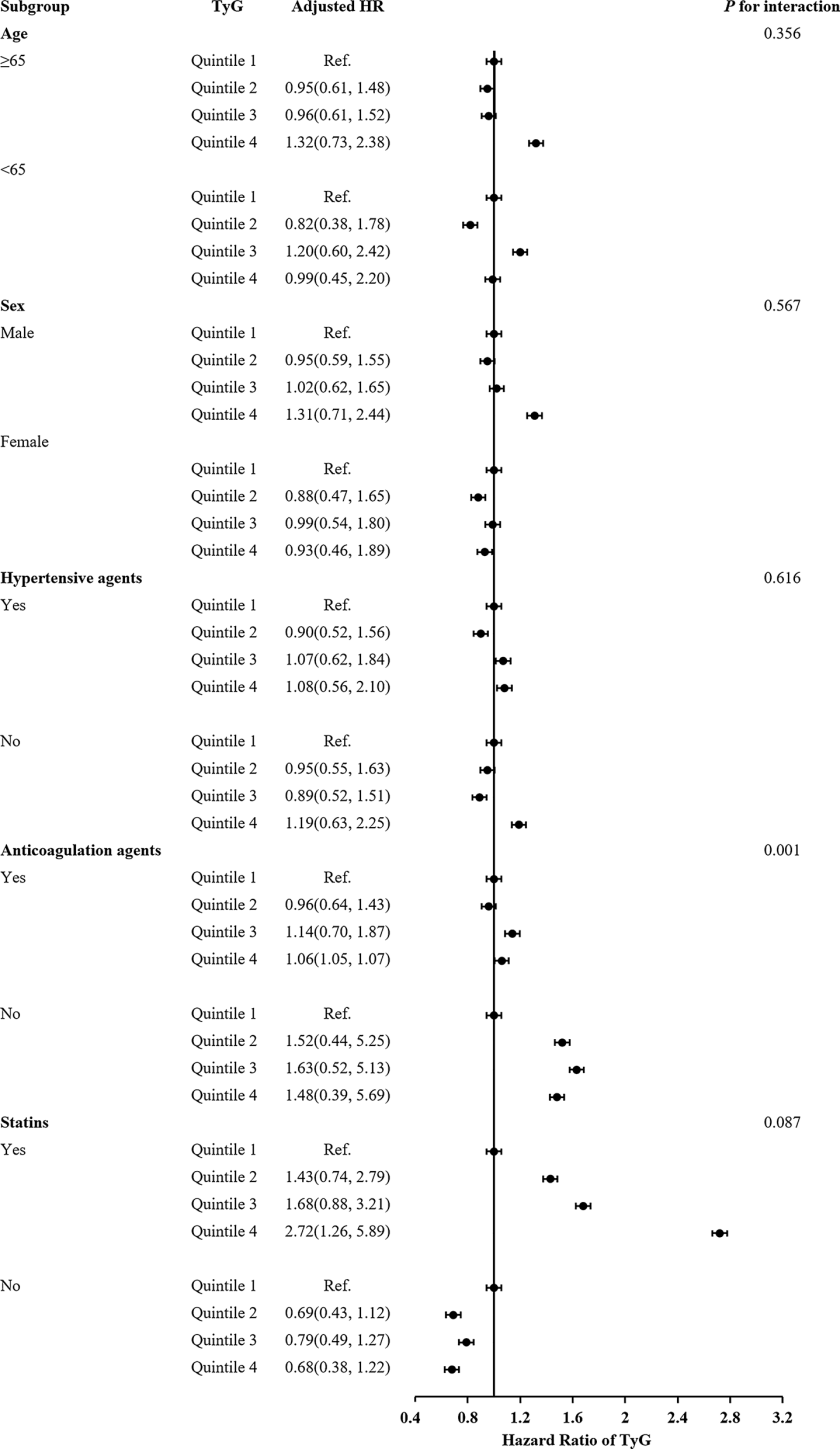
Forest plot of poor outcome in acute ischemic stroke patients with type-2 diabetes mellitus according to different subgroups. Adjusted model included age, sex, body mass index (BMI), systolic blood pressure (SBP), diastolic blood pressure (DBP), NIHSS on admission, Type A1C (HbA1c), total cholesterol (TC), triglyceride (TG), high-density lipoprotein cholesterol (HDL-C), low-density lipoprotein-C (LDL-C), fasting plasma glucose (FBG), intravenous thrombolysis, medical history of ischemic stroke, intracranial hemorrhage (ICH), atrial fibrillation, medication history of Statins, antidiabetics, antihypertension, medication at discharge of antidiabetic agents, statins, antihypertensive agents and TOAST subtypes

The subgroup analysis showed that there were interactions between TyG index and age (≥ 65), female, hypertensive agents, anticoagulant agents and statins in subgroup analyses especially anticoagulant drugs are very important for the prognosis of acute IS patients with T2DM (Figs. 3, 4, 5). Acute IS patient with T2DM received anticoagulant agents or not at discharge were associated with IS recurrence, all-cause death and poor outcome (*P* = 0.003, *P* = 0.006 and *P* = 0.001, respectively). And, patients with a higher TyG index without anticoagulant agents at discharge were more likely to have IS recurrence. Meanwhile, there were no interactions between the TyG index and age, sex, and hypertensive agents, statins at 1-year follow-up in subgroup analyses (all *P* values for interaction > 0.05).

## Discussion

In this study, we investigated the predictive role of the TyG index in ischemic stroke (IS) patients with type-2 diabetes mellitus (T2DM). This study, for the first time, demonstrated that a higher TyG index was a strong independent predictor of IS recurrence and all-cause mortality within 1 year after discharge. Even after adjustment for the association potential confounding risk factors, an independent association of the TyG index with adverse clinical outcomes remained. Therefore, TyG index deserves more attention in clinical practice and could be an independent prognostic factor in the acute IS patients with T2DM.

The TyG index, a novel indicator determined by TG and FBG, was demonstrated to be a useful predictor of metabolic disorders, T2DM, and atherosclerotic CV disease (CVD) [9, 17, 20]. During the past years, much work has been done to isolate the prediction of TyG index in CVD. Several studies implied high TyG index is associated with a higher risk of CVD. Lee et al. found a high TyG index can predict poor 3-month functional outcomes in patients with acute IS who undergo reperfusion therapy [21]. Yang et al. found elevated TyG index was associated with an increased risk of stroke recurrence and death [22]. Tai et al. showed that a higher levels of TyG index were significantly associated with an increased risk of major adverse cardiovascular events in patients with T2DM during 10 years follow-up [23]. A single-center, observational, retrospective cohort study including 1578 asymptomatic subjects with T2DM who underwent off-pump coronary artery bypass grafting (OPCABG) showed that TyG index may be a valuable predictor of adverse cardiovascular and cerebrovascular outcomes after OPCABG in patients with T2DM [24]. These studies suggested that TyG index can be a reliable predictor to predict the prognosis of cardiovascular and cerebrovascular related diseases.

However, some scholars thought the predict value of TyG index for clinical outcomes in acute IS patients remains limited and controversial [25, 26]. First, TyG index as a biomarker can be affected by hyperlipidaemia and hyperglycaemia. If patients with extremely high TG or FBG enrolled in clinical studies, TyG index may be not explore causality in cerebrovascular patients [27]. Second, the FBG and TG were more intuitive compared with TyG index and TyG index at baseline alone does not reflect the longitudinal association between the TyG index and cerebrovascular risk over time [25, 28]. Moreover, studies on TyG index are mostly focused on middle-aged and elderly individuals, and the value of TyG index is uncertainty in young subjects [29]. To allay these concerns, a few studies have provided the exact answer. As we all known, insulin resistance (IR) is closely related to obesity, hypertension, hyperlipidemia, as well as other metabolic syndrome (MetS) symptoms. HOMA-IR index, a means for detectingβ-cell function and IR, is widely used at present, but it has limited value in subjects receiving insulin treatment or those who do not have functioning beta cells [30]. To address this limitation, TyG index has been proven to be a reliable and accessible index for evaluating IR in high-risk individuals with and without diabetes by large clinical studies. A cross-sectional study involving 99 young and middle-aged individuals with various degrees of body weight and glucose tolerance was performed by Guerrero-Romero et al., and they found the TyG index as an optimal tool for the assessment of IR, showing high sensitivity (96.5%) and specificity (85.0%) compared to the gold standard, the hyperinsulinaemic-euglycaemic clamp test [31]. Another study by David et al. revealed that the TyG index had better predictive power (AUC: 0.75, 95% CI 0.7–0.81) in diagnosing subjects with DM than fasting blood glucose (FBG) measurement (AUC: 0.66, 95% CI 0.60–0.72) and TG levels (AUC: 0.71, 95% CI 0.65–0.77) among 4820 individuals [32]. That is to say, in some disease models, TyG is superior to single FBG or TG, which can better reflect the progress of the disease. Laura et al. first suggested a positive association between the TyG index (AUC: 0.708, 95% CI 0.68–0.73) and CVD events, including coronary heart failure (CHD), cerebrovascular disease, and peripheral arterial disease, independent of confounding factors [11]. So, TyG index can be a significant hallmark of different types of CVD, CAD and other metabolic related diseases.

The potential mechanism in the association of the TyG index with the development and progression of CVD in T2DM patients remains uncertain. TyG index is a surrogate marker of IR, led to the chronic inflammation, endothelium dysfunction, facilitated the formation of foam cells in the initiation of atherosclerosis [33–37]. Miao et al. found a higher TyG index as a promising atherosclerotic marker was associated with carotid atherosclerosis measured by carotid intima-media thickness in patients with IS [38]. Ahn et al. has shown TyG index consisting of lipid-related and glucose-related factors was a reliable marker of IR in the human body [39]. And, IR as a risk factor for CVD, it can lead to the development of CVD in both the general population and diabetes patients and predict the cardiovascular prognosis of patients with CVD [36]. Some studies have given the reason IR not only in atherogenesis but also in advanced plaque progression by promoting apoptosis of macrophages, endothelial cells, and vascular smooth muscle cells. First, TyG index can reflect the glucose metabolism, inflammation and oxidative stress in the body; Second, TyG index can reflect the metabolism of glycosylation products, oxygen radical, nitric oxide (NO) and platelet reactivity, which can lead to endothelial cell-dependent vasodilation [40]. Guo et al. found the elevation of the TyG index is associated with enhanced platelet reactivity and higher prevalence of aspirin high residual on-treatment platelet reactivity (HRPR) and could be an independent risk factor for aspirin HRPR with IR assessment [17]. Finally, increased TyG levels can increase the level of free fatty acids (FFA) and FFA flux from adipose to non-adipose tissues, which may accompany IR. And, lowering TyG levels appear to be an additional target in patients with a high CVD risk [41–44]. And, in our study, the characteristics of the population in our study evaded the restriction of TyG index and we found patients with a high TyG index were more likely to have higher BMI, TC, LDL-C, and have lower HDL-C, suggesting that the observed association between the TyG index and poor prognosis may be explained by the presence of cardiovascular risk factors. According to the above reasons, it can be explained why the TyG index has an association with IS recurrence and all cause death in acute IS patient with T2DM at 1 year follow-up in our study. These findings are partially in consist with previous findings in non-T2DM patients with acute IS. Patients with a higher TyG index were more likely to have a higher IR and more pronounced metabolic disorders including higher BMI, increased waist circumference, TC, TG, LDL-C, non-HDL-C, plasma glucose and HbA1c levels, lower HDL-C levels and also less physical activity [6, 45, 46]. These risk factors may contribute to IS recurrence and all-cause death of IS in T2DM patient.

In this study, we further explored the effect of age, sex, and hypertensive agents, anticoagulant agents, statins on the predictive performance of TyG index for adverse clinical outcomes. We conducted a correlation analysis among the enrolled patients. Form the result, we can see patients received anticoagulant agents at discharge or not have a statistically significance in IS recurrence, all-cause death and poor outcome at 1 year follow-up. Patients with higher TyG index without anticoagulant agents more likely to have IS recurrence. In response to the above results, previous researches have given the reason. Li et al. found aspirin combined with clopidogrel/clopidogrel active metabolite (CAM) or ticagrelor could reduce inflammasome mediated pyroptosis in middle cerebral artery occlusion/reperfusion (MCAO/R) rats and oxygen glucose deprivation/reperfusion (OGD/R) PC12 cells through NF-κB/NLRP3 signaling pathway [47]. Hankey et al. found the combination of dipyridamole plus aspirin is the preferred antiplatelet regimen to reduce the risk of recurrent vascular events among patients with TIA and ischemic stroke of arterial origin [48]. Kirshner et al. found antiplatelet therapy especially aspirin, with extended-release dipyridamole (ER-DP) is effective in secondary stroke prevention [49]. So, anticoagulant therapy, especially antiplatelet therapy after IS, is an important measure to improve the prognosis of IS patient with T2DM.

Our study confirmed that TyG index could be used as an effective predictor in clinical practice, and was an independent risk predictor of IS recurrence and all-cause death. However, there are several limitations in our study. First, patients included in our study were all Chinese patients, who are known to have a higher rate of large artery atherosclerosis disease associated with a higher risk of stroke recurrence [50]. And, the applicability of our findings to other ethnic groups is uncertain. Second, only baseline TyG index was available in our study. Dynamic changes of TyG index during three months and one year time follow-up was not available. So, we could not analyze the causality of the indicators in patients with the time of recurrent IS. Third, baseline characteristics between patients included and not included in our study were not entirely balanced. In particular, the history time of IS patient with T2DM and hyperlipidemia was not analyzed. Fourth, follow-up information was only by telephone in this study, and end point assessment could not be validated by hospital records in all individuals. Further large-scaled cohort studies in other populations are needed to verify the generalizability of our findings.

## Conclusions

Our results strongly suggested that TyG index may be used as a prominent risk predictor to assess the long-term outcomes in IS patients with T2DM. Elevated levels of TyG index can be used an indicator of increased risk of recurrent IS and all-cause death at 1 year follow-up. Monitoring the TyG index as a qualified predictor and risk stratification tool deserves more attention in clinical practice for IS patients with T2DM.

## Supplementary Information

**Additional file 1: Table S1.** Pearson correlation between TyG index and HBG, FBG, TC, TG, HDL, LDL in in acute ischemic stroke patients with type-2 diabetes mellitus included in this analysis.

**Additional file 2: Table S2.** HRs (95% CIs) for risk of events in pre-diabetes patients with acute ischemic stroke according to TyG index quartiles in this study.

## Data Availability

All data generated or analysed during this study are included in this published article. The datasets used and/or analysed during the current study are available from the corresponding author (Dr. Yilong Wang) on reasonable request.

## Abbreviations

TyG: The triglyceride-glucose
IS: Ischemic stroke
T2DM: Type-2 diabetes mellitus
CNSRII: China National Stroke Registry II
TG: Triglycerides
FBG: Fasting plasma glucose
MI: Myocardial infarction
TIA: Transient ischemic attack
BMI: Body mass index
SBP: Systolic blood pressure
DBP: Diastolic blood pressure
TG: Triglyceride
HDL: High-density lipoprotein cholesterol
LDL: Low-density lipoprotein
ICH: Intracerebral hemorrhage
AF: Atrial fibrillation
CS: Carotid stenosis
