# Supplementary material for: Predictive effect of triglyceride-glucose index on clinical events in patients with acute ischemic stroke and type 2 diabetes mellitus
Source: Cardiovasc Diabetol. 2022 Dec 12;21:280. doi: 10.1186/s12933-022-01704-4 (PMC9743618; doi:10.1186/s12933-022-01704-4)
Supplement: Supplementary file 1 — Additional file 1: Table S1. Pearson correlation between TyG index and HBG, FBG, TC, TG, HDL, LDL in in acute ischemic stroke patients with type-2 diabetes mellitus included in this analysis. [file 12933_2022_1704_MOESM1_ESM.doc]

**Table S1.** Pearson correlation between TyG index and HBG, FBG, TC, TG, HDL, LDL in in acute ischemic stroke patients with type-2 [diabetes mellitus](https://www.baidu.com/link?url=XIlwWhQYjDqku-FxAWDd1y7hjgiXCOEjPxeYzKyalwh0FxywjI4pEZl0U8mvfZmuvAHqKyzfcxw94PemZ1vMButRwp7VlK9vfiJMLbkRaF6oKF-o4SElufz8M2_CfaHn&wd=&eqid=92e4bb4900215b110000000362a334f7) included in this analysis

| **Variables** | **r** | ***P*value** |
| --- | --- | --- |
| HbA1c | 0.337 | <0.001 |
| FBG | 0.588 | <0.001 |
| TC | 0.333 | <0.001 |
| TG | 0.751 | <0.001 |
| HDL | -0.092 | <0.001 |
| LDL | 0.202 | <0.001 |

HbA1c, Glycosylated Hemoglobin, Type A1C; TC, total cholesterol; TG, triglyceride; HDL-C, high-density lipoprotein cholesterol; LDL-C, low-density lipoprotein-C; FBG, fasting plasma glucose.
