# Supplementary material for: Predictive effect of triglyceride-glucose index on clinical events in patients with acute ischemic stroke and type 2 diabetes mellitus
Source: Cardiovasc Diabetol. 2022 Dec 12;21:280. doi: 10.1186/s12933-022-01704-4 (PMC9743618; doi:10.1186/s12933-022-01704-4)
Supplement: Supplementary file 2 — Additional file 2: Table S2. HRs (95% CIs) for risk of events in pre-diabetes patients with acute ischemic stroke according to TyG index quartiles in this study. [file 12933_2022_1704_MOESM2_ESM.doc]

**Table S2.** HRs (95% CIs) for risk of events in pre-diabetes patients with acute ischemic stroke according to TyG index quartiles in this study.

|  | **Events, n (%)** | **Adjusted HR*** | ***P* value** | **Adjusted HR†** | ***P*value** | **Adjusted HR§** | ***P*value** |
| --- | --- | --- | --- | --- | --- | --- | --- |
| **1 year follow-up** |  |  |  |  |  |  |  |
| **Ischemic stroke recurrence** |  |  |  |  |  |  |  |
| Quintile 1 | 54 (8.35) | Ref. |  | Ref. |  | Ref. |  |
| Quintile 2 | 47 (7.25) | 0.99(0.67, 1.47) | 0.978 | 1.06(0.70, 1.60) | 0.781 | 1.03(0.68, 1.56) | 0.876 |
| Quintile 3 | 42 (6.49) | 0.95(0.63, 1.43) | 0.793 | 1.05(0.66, 1.68) | 0.823 | 1.05(0.66, 1.67) | 0.835 |
| Quintile 4 | 37 (5.71) | 0.92(0.60, 1.41) | 0.695 | 0.86(0.46, 1.60) | 0.632 | 0.90(0.49, 1.64) | 0.721 |
| **All-cause death** |  |  |  |  |  |  |  |
| Quintile 1 | 58 (8.96) | Ref. |  | Ref. |  | Ref. |  |
| Quintile 2 | 44 (6.79) | 0.95(0.64, 1.40) | 0.785 | 1.16(0.74, 1.82) | 0.507 | 1.13(0.73, 1.76) | 0.586 |
| Quintile 3 | 40 (6.18) | 1.00(0.66, 1.51) | 0.995 | 1.44(0.80, 2.59) | 0.220 | 1.42(0.81, 2.51) | 0.220 |
| Quintile 4 | 21 (3.24) | 0.62(0.38, 1.04) | 0.070 | 1.16(0.43, 3.09) | 0.767 | 1.08(0.43, 2.70) | 0.875 |
| **Poor outcome** |  |  |  |  |  |  |  |
| Quintile 1 | 163 (26.42) | Ref. |  | Ref. |  | Ref. |  |
| Quintile 2 | 142 (23.39) | 0.99(0.76, 1.30) | 0.959 | 0.87(0.64, 1.19) | 0.391 | 0.87(0.63, 1.19) | 0.375 |
| Quintile 3 | 120 (20.07) | 0.89(0.67, 1.18) | 0.405 | 0.75(0.53, 1.08) | 0.124 | 0.78(0.54, 1.13) | 0.189 |
| Quintile 4 | 92 (15.03) | 0.72(0.53, 0.98) | 0.035 | 0.58(0.35, 0.97) | 0.038 | 0.66(0.39, 1.11) | 0.016 |

*Adjusted 1, for variables of age and sex.

†Adjusted 2, adjusted 1+ body mass index (BMI), systolic blood pressure (SBP), diastolic blood pressure (DBP), NIHSS on admission, Glycosylated Hemoglobin, Type A1C (HbA1c), total cholesterol (TC), triglyceride (TG), high-density lipoprotein cholesterol (HDL-C), low-density lipoprotein-C (LDL-C), fasting plasma glucose (FBG).

§Adjusted 3, adjusted 2 + intravenous thrombolysis, medical history of ischemic stroke, intracranial hemorrhage (ICH), atrial fibrillation (AF), medication history of Statins, antidiabetics, antihypertension, medication at discharge of antidiabetic agents, statins, antihypertensive agents and TOAST subtypes.

Poor functional outcomes were defined as a modified Rankin Scale score of 3-6.
